# Developing an Oral Hygiene Education Song for Children and Teenagers in Nigeria

**DOI:** 10.1016/j.identj.2022.06.008

**Published:** 2022-07-30

**Authors:** Olushola Ibiyemi, Folake Lawal, Mary Osuh, Tolu Owoaje, Ejiro Idiga, Omotayo Fagbule, Olabode Ijarogbe

**Affiliations:** a Department of Periodontology and Community Dentistry, University College Hospital, Ibadan, Nigeria; b Department of Periodontology and Community Dentistry, Faculty of Dentistry, College of Medicine, University of Ibadan, Nigeria; c Department of Music, University of Ibadan, Nigeria; d Department of Restorative Dentistry, Faculty of Dental Sciences, College of Medicine, University of Lagos, Lagos, Nigeria

**Keywords:** Educational intervention, Adolescents, School, Students, Dental health, Folk song

## Abstract

**Background:**

Oral diseases mainly caused by poor oral hygiene are a major public health problem affecting over half of the world's population. Oral hygiene education targeted at children and teenagers in schools is an important approach in addressing this problem. Folk songs in the language and cultural context children and teenagers are familiar with appears to be a promising tool and alternative to traditional oral hygiene education.

**Objectives:**

This paper aims to report how a local traditional song on oral hygiene education amongst children and teenagers in southwestern Nigeria was developed with a view to providing information on how the song can be developed in other languages as well as how other oral health education songs can be developed.

**Method:**

Oral health professionals from the University College Hospital, Ibadan, and music experts from the University of Ibadan, in collaboration with traditional/local musicians, parents/guardians, schoolteachers, and community heads, took part in the development of the song over a period of 6 months. Developing the tool involved certain processes which were validated and evaluated. These processes included developing the lyrics, choosing the type of song, creating the melody, and producing and finishing the song as an oral hygiene education tool. Written and audio documentations of the processes were done.

**Results:**

A 90-second oral hygiene education song was developed in English and later translated into Yoruba. The numerous steps, collaborations, and meetings required in the development of the song were associated with many learning opportunities including team-building, understanding cultural contexts, effective collaboration, leadership, and communication skills.

**Conclusions:**

Creating new and effective oral hygiene education tool requires various processes and multiple steps and resources. However, it is a necessary and worthy exercise in ensuring sustainable and adequate oral hygiene, especially amongst children and teenagers in underserved populations, as we move into the future.

## Introduction

Oral disease is a major public health problem, and it is estimated to affect 3.5 billion people worldwide.^1^, ^2^, ^3^ Poor oral hygiene is a major cause of dental caries and periodontal diseases, the 2 most common oral diseases. The world over, the prevalence of dental caries and periodontal diseases is high, affecting nearly 100% in some populations under survey in many countries for diseases that are very amenable to prevention and control.^4^ In Nigeria, the prevalence of dental caries and periodontal diseases ranges between 4% and 30% and 15% and 94%, respectively.^5^^,^^6^ Dental caries and periodontal diseases are preventable by reducing dietary sugar consumption and improving oral hygiene. Childhood, especially the first years of life, is the time when proper health-promoting behaviours are being formed, allowing the future prevention of such diseases such as dental caries and periodontal diseases.^7^ Promoting oral health amongst children and young people is essential to improving their oral health. Individual characteristics and behaviour are important aspects of focus as determinants of health. Further, recent evidence showed that oral health awareness amongst children and adolescents is very low, and this is a source of concern in low-income countries.^8^^,^^9^ Dental health education targeted at students is an important approach in addressing this problem. Educating children and younger people can create healthy behaviours for life. Improving children's oral hygiene behaviours should help reduce dental caries and periodontal diseases and their associated health burden. Habits are easier to form and appear to last longer at this developmental phase, with the consequent effects of these habits impacting the individual's quality of life for the rest of their life.^10^ This is even more important as studies suggest that children in the lower socioeconomic class have poorer oral hygiene practices and poorer oral health in Nigeria and many African countries.^11^, ^12^, ^13^

Traditionally, oral hygiene education is carried out by dentists or other dental care professionals using lectures.^14^^,^^15^ However, there appear to be some limitations in using traditional methods, including a shortage of dentists to ensure sustainability; limitations in reaching a large proportion of the population; the didactic nature of traditional oral hygiene education, which could make it boring and unmemorable; and limitations due to lack of cultural relevance and relatedness to the audience.^5^^,^^15^ There is a need to strengthen oral hygiene promotion strategies and to apply of new health education tools in the face of these challenges.^16^ A viable alternative is the use of music.

Oral health and music have always had an intricate relationship, especially in the traditional African setting.^7^ In Africa, music forms an integral part of everyday life. It inspires the African from the morning hours, through the day and till night. It is a part of his birth, growth, and development throughout life and even after death.^7^ Beliefs and practices pertaining to everyday living, such as eating, oral hygiene, and social norms, are often effectively communicated through songs in the traditional African setting.^17^ Songs on oral hygiene practices can be a great way to have children and teenagers learn and remember how to practice adequate oral health.^18^ When children and teenagers learn through music, they might not even realise they are having fun whilst setting healthy lifetime habits. Toothbrushing songs set to familiar tunes make learning about oral health fun and easy.^7^ Tunes of traditional songs can be combined with words specifically adapted to make the song connect to a teachable concept.^18^ Parents, guardians, and schoolteachers can use songs as a fun way to teach brushing and good oral hygiene. Using songs as a reminder that it is time to brush teeth is something parents/guardians and their children/wards can do together each day.

There have been no reports documenting music developed in a local Nigerian language as an oral hygiene education tool. This paper describes the rationale behind the choice of a culturally appropriate oral hygiene education song and the process that went into the finished work. Developing such a tool for oral hygiene education draws on many core public health competencies such as leadership, advocacy, critical analysis, communication, community–cultural orientation, management, and professionalism and ethics.^19^

Therefore, the aim of this paper is to report how a local traditional song on oral hygiene education amongst children and teenagers in southwestern Nigeria was developed and to provide information on how the song can be developed in other languages and how other oral health education songs can be developed.

## Background

A 90-second oral hygiene education song was developed in English and later translated into Yoruba, a major Nigerian language spoken mainly by inhabitants of southwestern Nigeria. The song was developed to be used as a tool for health education on oral hygiene practices amongst children and teenagers in southwestern Nigeria. The goal of the song is to improve the oral hygiene of children and teenagers by educating them on the importance of good oral hygiene and proper oral hygiene practices (proper toothbrushing method, twice-daily toothbrushing, and the use of an adequate amount of fluoride-containing toothpaste) in a way that is fun and memorable. Music presents a unique opportunity to inform whilst entertaining.^20^ Music in a child's own culture is an ideal tool for her education, as the child will respond with joy, delight, and interest.^17^^,^^20^ In low-income countries, several projects have used oral health education inform of lectures, pamphlets, and videos to improve children's oral health, but not much has been achieved because many of the interventions were not developed in consultation with local stakeholders, some did not incorporate familiar cultural elements, and others required the audience to be able to read.^21^ A song developed in conjunction with parents, guardians, and schoolteachers and set to tunes and language that children and teenagers will be familiar with should improve their oral hygiene. The song, if effective, could be adopted by people of other tribes and ethnic groups.

Some of the challenges experienced in the development of this tool were addressed in this paper. These include challenges associated with selecting and working with the team involved in developing the tool, developing the lyrics and the melody, validating the tool, and production and finishing of the tool.

## Creating the music tool for oral hygiene education

Research findings in Nigeria showed that poor oral hygiene is a major public health problem amongst children and teenagers particularly, in lower social classes.^11^^,^^22^ Oral health professionals and music experts in the University of Ibadan and University College Hospital, Ibadan, southwestern Nigeria, as well as traditional/local musicians, parents/guardians, schoolteachers, and community heads collaborated to produce an oral hygiene education song. Creating the song in both English and Yoruba as an oral hygiene education tool involved a series of processes.

## The team

The development of the tool was achieved by a team comprising dental public health professionals, a professional music educator, and contemporary and traditional/local musicians. There was also input from children, teenagers, parents/guardians, schoolteachers, and community heads.

Meetings, seminars, and sessions where discussions, consultations, and reviews were made constituted essential parts in developing the tool. This led to challenges associated with selecting an appropriate time and location convenient for everyone involved in the project. However, these challenges were addressed by conducting some of these sessions online via Zoom. The online platform also facilitated the ease with which the song's audio files were shared, listened to, and commented on.

## Developing the song

There is no one way to develop a song.^23^ In developing this present song, various factors were taken into consideration. These included the type of song, the message in the song (lyrics), the melody, local musical idiom, the duration of the song, and the memorability of the song.

## Creating the type and lyrics of the song

The dental public health professionals from the University of Ibadan and University College Hospital Ibadan had 3 meetings where the type and lyrics of the oral hygiene song were agreed on. It was agreed that the oral hygiene song should first be developed in English and then translated into Yoruba. The lyrics should focus on oral hygiene messages on toothbrushing method and frequency and using an adequate amount of fluoride-containing toothpaste. The dental health professionals then had 3 other meetings with a music expert from the University of Ibadan to further develop the lyrics of the song. The lyrics contained concise oral hygiene messages that can improve oral hygiene knowledge, attitude, and practice amongst children and teenagers and spur them to action. The words of the lyrics were further simplified to ensure that it was relevant to children's and teenagers’ age and life experiences. The songs were broken down into short lines and verses to ensure that they were easy to learn and memorable. Two versions were created: one in the English language and the other in Yoruba. The English and Yoruba songs could not share exactly the same melodic, syllabic, and poetic properties because the Yoruba language is tonal and possesses unique musical properties. Moreover, English songs translated to the Yoruba Language do not make any linguistic sense if they are sung to the same melody as the English song. As Yip (2002) notes,^24^ if the pitch of a word can change its meaning, not just its nuances but its core meaning, the word comes from a tone language.

This then necessitated the development of original lyrics for the Yoruba song within the framework of the English song texts. Dental public health professionals carried out content and construct validation of the lyrics. These were undertaken by asking 2 paediatric dentists, 6 primary and 5 secondary schoolteachers, 7 parents/guardians, 12 children and teenagers, and 2 community leaders to read the lyrics of the messages and write down their observations on a sheet of paper given to them. Observations such as long song duration (180 seconds) and unclear messages were made. These observations were resolved by reducing the songs’ duration to 90 seconds and making the messages simple and clear. Evaluation of the lyrics was then carried out to ensure it met the specified requirement for oral hygiene promotion.

## Developing the melodies

The development of the melody was carried out by the music educator from the University of Ibadan and professional musicians. Samples from common children's rhymes, contemporary songs, and local and traditional songs were used as guide in developing the tunes. Most especially, the indigenous melodic properties of Yoruba songs were taken into consideration in composing the melody of the Yoruba song. This is in order to ensure that the song identifies with the musical experience of the target population. First, the composition of the indigenous song follows the rhythmic nature of Yoruba music, which is exemplified in the *konkolo* timeline pattern which features predominantly in Yoruba music.^25^ As shown below, the first bar illustrates the *konkolo* rhythm of the Yoruba, whilst the second bar illustrates a complex variant of the same timeline pattern. The rhythmic framework is situated in compound quadruple time signature.

**Figure:**
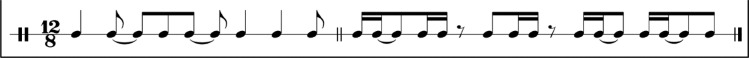

Composition of the Yoruba song followed the speech tone correlation in the song text and the melody, owing to the tonal nature of the Yoruba language, wherein song melody has to align with the speech tone in order to retain linguistic meaning. Shown below is the musical score of the English song.

**Figure:**
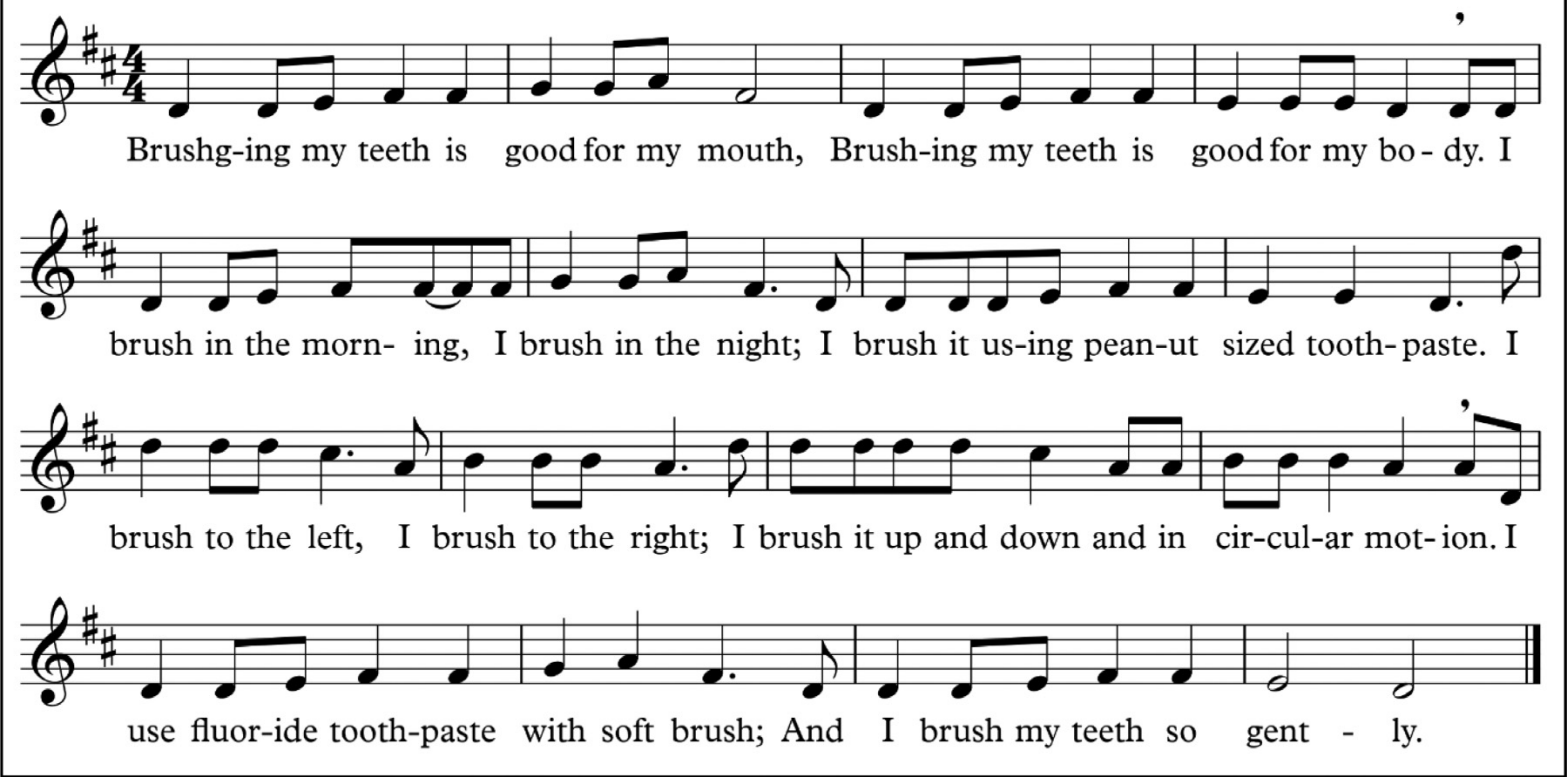

Brushing my teeth is good for my mouth

Brushing my teeth is good for my body

I brush in the morning, I brush in the night

I brush it using peanut-sized toothpaste

I brush to the left, I brush to the right

I brush it up and down and in circular motion

I use fluoride toothpaste with soft brush

And I brush my teeth so gently

This song was composed in phrases and structured in simple ternary form. Shown below is the musical score of the Yoruba song, which shares thematic elements with the English song from which it was translated.

**Figure:**
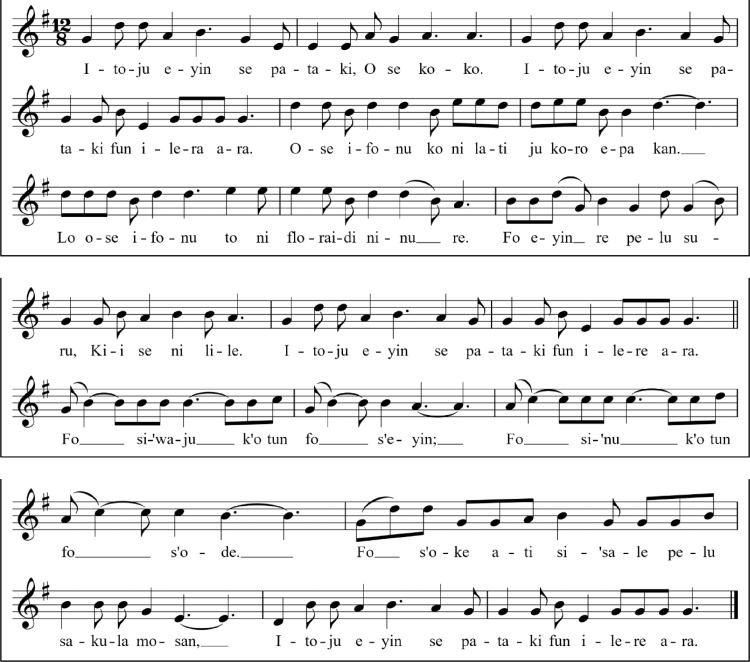

**Table:** 

*Itoju eyin se pataki o se koko*	Caring for the teeth is important
*Itoju enu se pataki fun ilera ara*	Caring for the teeth is important for healthy living
*Ose ifonu ko ni lati ju koro epa kan*	Brush with peanut-sized toothpaste
*Lo ose ifonu to ni floraidi ninu re*	Make use of fluoride toothpaste
*Fo eyin re pelu suuru, kii se ni lile*	Brush your teeth very gently
*Itoju eyin se pataki fun ilera ara*	Caring for the teeth is important
*Fo o siwaju*	Brush it to the front
*K'o tun fo o s'eyin*	And brush it to the back
*Fo o sinu k'o tun fo o s'ode*	Brush it in and out
*Fo o soke ati si'sale pelu sakula mosan*	Brush it up and down and in circular motion
*Itoju eyin se pataki fun ilera ara*	Caring for the teeth is important

The song was composed in phrases and structured in solo and chorused refrain in order to ensure sing-ability and memorability. The notation of the song was done using a standard staff notation system, on the key of G major and compound quadruple time signature. Several Yoruba songs were created as tool for this study, but only one which possesses the most appropriate properties needed for this study was selected.

## Production

The tools were taken to a music recording studio for production and easy accessibility and dissemination. Appropriate beats inspired by popular and traditional/local musical instruments were added to the song. Professional singers were engaged in the recording of the song. The English song was recorded using the voice texture of schoolchildren and teenagers in order to situate it within their musical imagination and make the target population personalise the lessons therein. The songs were thereafter mixed and mastered by a music production professional.

## Validating and evaluating the tools

Following the production of the first version of the tools, face, content, and construct validity of the tool was assessed amongst 2 dental public health professionals and 2 music educators who could read and write in both English and Yoruba. The songs were sent to WhatsApp on their smartphones, where they played and listened to the songs. They were asked to write their comments and observations on a sheet of paper given to them. Further validation was carried out amongst 10 schoolchildren and teenagers, 8 parents of students, 5 primary school and 6 secondary school teachers, and 3 community leaders. They were also asked to write their comments and observations on a sheet of paper given to them. Some of the important aspects of the tool that were validated include the duration, ease of learning, ease of singing, likability, memorability, and relevance of the song to the students’ life experiences and age. Data obtained from the validation exercise were analysed by a data analyst and the dental public health professionals. Modifications where then made on the song in order to improve its usefulness as an oral hygiene education tool. After modifications were made, evaluation of the song was carried out by having students, teachers, and community leaders listened to the song again and give feedback. They agreed that the song was suitable and adequate for use in promoting oral hygiene amongst children.

## Finalising the tools

The music tools for oral hygiene education were subsequently taken back to the studio to effect the changes made and produce the final version.

## Future implications

The goal of this project was to produce an oral hygiene educational song that can be used to improve the oral hygiene knowledge, attitude, and practice of children and teenagers. In addition, the effectiveness of the song on oral hygiene will be measured amongst schoolchildren and teenagers. After evaluating the effectiveness of the songs, advocacy will be made to the Ministry of Education for their incorporation into the curriculum of schools as oral health education tools. The songs will also be used in oral health outreach amongst children and teenagers. The process of developing both songs will provide empirical data that can be built on in developing other health educational tools.

This project has helped in improving the competence of the dental public health professionals involved in the project in creating oral health education tools, collaborating with various disciplines, and building relationships with different groups, which is necessary for the future of public health interventions.

## Learning points

The process of developing both songs as oral hygiene education tools availed numerous learning points. First, leadership was a core aspect of the process, as team members had to attend meetings to interact and learn from each other as well as deliver on assignments within the stipulated timeline. Sub-team leaders were selected and trained to lead the various sub-teams formed through the different stages. Second, the communication skills of all involved in the project were built upon, as smooth and effective interactions were paramount between different individuals from different fields as well as with teachers, schoolchildren, parents, and community heads. Third, understanding the cultural relevance of the content and context of messages in music was a learning point also. Although, the COVID-19 pandemic caused some setbacks in the timeline for this project, they were successfully overcome by having a mix of online and onsite meetings when necessary. The entire process involved many positive and successful outcomes such as building healthy relationships, professionalism and ethical conduct amongst the professionals from multiple disciplines involved in the project, and successfully creating and validating the oral hygiene education tool.

## Conclusions

Oral diseases continue to be a burden amongst children and teenagers, especially in underprivileged and underserved communities. Exploring new and effective tools, like the use of music, for promoting oral hygiene education amongst children and teenagers is a necessity. Music is a vital part of the life of the African and can consequently be used to mould the behaviour of individuals, especially in their developing schooling years.

Developing new and effective oral hygiene education tools requires a variety of resources, techniques, and steps. However, it is a necessary and worthwhile activity in ensuring the long-term maintenance of good oral health, particularly amongst children and teenagers, as we progress into the future.

## Conflict of interest

None disclosed.
